# The Plasmodesmal Localization Signal of TMV MP Is Recognized by Plant Synaptotagmin SYTA

**DOI:** 10.1128/mBio.01314-18

**Published:** 2018-07-10

**Authors:** Cheng Yuan, Sondra G. Lazarowitz, Vitaly Citovsky

**Affiliations:** a Department of Biochemistry and Cell Biology, State University of New York, Stony Brook, New York, USA; b Tobacco Breeding and Biotechnology Research Center, Yunnan Academy of Tobacco Agricultural Sciences, Kunming, Yunnan, China; c Department of Plant Pathology and Plant-Microbe Biology, Cornell University, Ithaca, New York, USA; University of California, Berkeley

**Keywords:** plasmodesmata, movement protein, targeting, *Tobacco mosaic virus*

## Abstract

Plant viruses cross the barrier of the plant cell wall by moving through intercellular channels, termed plasmodesmata, to invade their hosts. They accomplish this by encoding movement proteins (MPs), which act to alter plasmodesmal gating. How MPs target to plasmodesmata is not well understood. Our recent characterization of the first plasmodesmal localization signal (PLS) identified in a viral MP, namely, the MP encoded by the *Tobamovirus Tobacco mosaic virus* (TMV), now provides the opportunity to identify host proteins that recognize this PLS and may be important for its plasmodesmal targeting. One such candidate protein is *Arabidopsis* synaptotagmin A (SYTA), which is required to form endoplasmic reticulum (ER)-plasma membrane contact sites and regulates the MP-mediated trafficking of begomoviruses, tobamoviruses, and potyviruses. In particular, SYTA interacts with, and regulates the cell-to-cell transport of, both TMV MP and the MP encoded by the *Tobamovirus Turnip vein clearing virus* (TVCV). Using *in planta* bimolecular fluorescence complementation (BiFC) and yeast two-hybrid assays, we show here that the TMV PLS interacted with SYTA. This PLS sequence was both necessary and sufficient for interaction with SYTA, and the plasmodesmal targeting activity of the TMV PLS was substantially reduced in an Arabidopsis syta knockdown line. Our findings show that SYTA is one host factor that can recognize the TMV PLS and suggest that this interaction may stabilize the association of TMV MP with plasmodesmata.

## IMPORTANCE

Plant viruses use their movement proteins (MPs) to move through host intercellular connections, plasmodesmata. Perhaps one of the most intriguing, yet least studied, aspects of this transport is the MP signal sequences and their host recognition factors. Recently, we have described the plasmodesmal localization signal (PLS) of the *Tobacco mosaic virus* (TMV) MP. Here, we identified the *Arabidopsis* synaptotagmin A (SYTA) as a host factor that recognizes TMV MP PLS and promotes its association with the plasmodesmal membrane. The significance of these findings is two-fold: (i) we identified the TMV MP association with the cell membrane at plasmodesmata as an important PLS-dependent step in plasmodesmal targeting, and (ii) we identified the plant SYTA protein that specifically recognizes PLS as a host factor involved in this step.

## INTRODUCTION

Plasmodesmata (Pd) are complex plant transwall pores that serve as gateable channels for the cell-to-cell trafficking of endogenous macromolecules and invading viral pathogens (1–5). Historically, the first virus shown to traffic through Pd was the *Tobamovirus Tobacco mosaic virus* (TMV), which encodes a single 30-kDa viral protein termed movement protein (MP) (6). The major activities of TMV MP associated with its ability to mediate virus spread between cells include binding to single-stranded nucleic acids and viral genomes, targeting to Pd, and increasing Pd permeability to promote intercellular viral genome transport (7–10). Despite intensive investigation of TMV MP (6, 7, 9), the molecular pathway for its targeting to Pd remains largely undefined.

Sorting of proteins into the correct targeting pathway and ultimately to the proper subcellular compartment or extracellular space requires that appropriate receptor molecules recognize specific targeting signals within the cargo protein. For example, importins interact with basic nuclear localization signals in order to target specific cargo proteins to the nuclear pore via an importin-alpha-dependent pathway (11, 12). We recently identified the first plasmodesmal localization signal (PLS) in a viral MP, located within the N-terminal 50 amino acid residues of the TMV MP (MP^1–50^) (13). This now allows us to identify host cell proteins that recognize MP^1–50^ and which may facilitate TMV MP targeting to or association with Pd. One such candidate protein is *Arabidopsis* synaptotagmin A (SYTA), which is required to form endoplasmic reticulum-plasma membrane (ER-PM) contact sites in Arabidopsis thaliana (14). SYTA has been shown to regulate the ability of diverse viral MPs, including TMV MP and the MP encoded by the related *Tobamovirus Turnip vein clearing virus* (TVCV), to alter Pd to promote virus cell-to-cell transport (14, 15). Specifically, TVCV MP interacts with SYTA at ER-PM contact sites to remodel these sites during virus infection. This remodeling forms virus replication sites at Pd and relocates SYTA to within Pd active in MP cell-to-cell transport. Importantly, SYTA at ER-PM contact sites is required for TVCV MP to accumulate in Pd during infection (14). We now show here that SYTA interacted with TMV MP^1–50^. The PLS sequence in MP^1–50^ was both necessary and sufficient for this interaction with SYTA, and the accumulation of TMV MP^1–50^ at Pd, like that of TVCV MP, was reduced by nearly 3-fold in an Arabidopsis syta knockdown line. Thus, SYTA appears to be a host cell factor that can recognize the PLS in TMV MP^1–50^ and stabilize MP association with Pd.

## RESULTS

### TMV MP^1–50^ interacts with *Arabidopsis* SYTA.

We expected that a PLS-interacting protein involved in Pd targeting would possess at least two important features: it would be located in close proximity to the plasma membrane, in which Pd are known to reside (16), and it would be known to interact with full-length TMV MP and be required for MP intercellular movement. It might also be associated with the ER, with which the MPs encoded by TMV and TVCV are also known to associate (17). In particular, both of these MPs associate with cortical ER membrane to form virus replication sites adjacent to Pd (18–20). Among all identified TMV MP-interacting proteins, only one fulfills all three criteria, namely, *Arabidopsis* SYTA (14, 15). We therefore examined whether SYTA interacted with TMV MP^1–50^ and whether this interaction was impaired in either of the two known single-amino-acid-substitution mutants in the PLS that do not target to Pd, MP^1–50(V4A)^ and MP^1–50(F14A)^ (13). Notably, although there is about 58% conservation between the amino acid sequence of the TMV MP^1–50^ and the corresponding regions of other *Tobamovirus* MPs, the analogous Phe-14 is found in all *Tobamovirus* MPs, and TVCV MP also has Val at amino acid position 4, with Val or the conservative substitution Met being found at the equivalent position in other *Tobamovirus* MPs (18).

**FIG 1:**
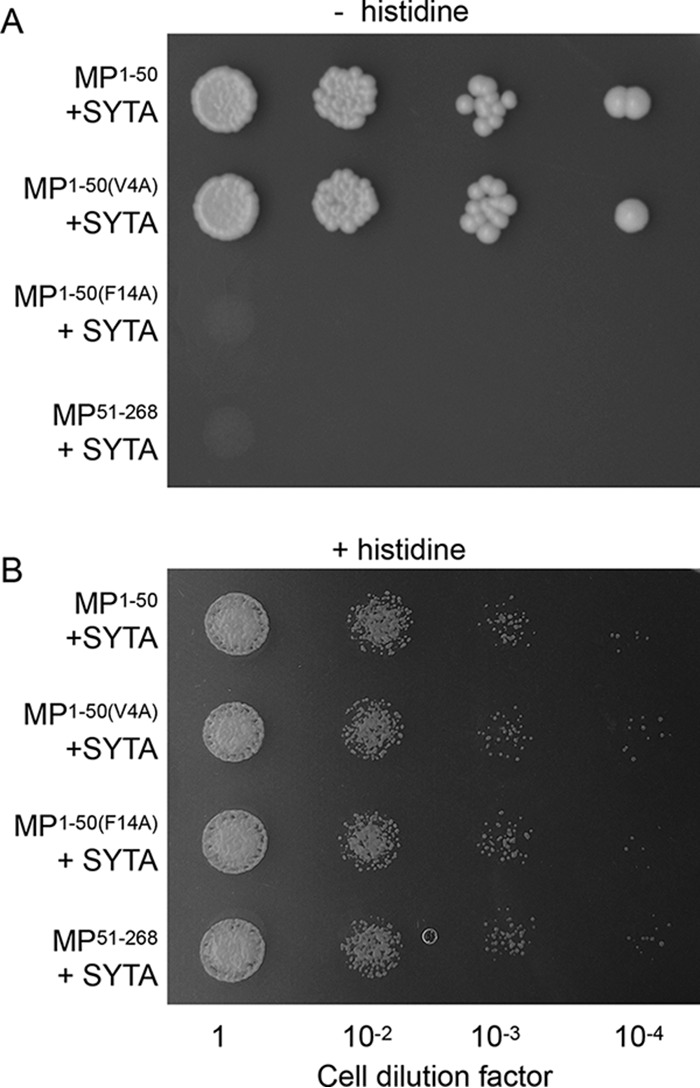
MP^1–50^-SYTA interaction in the yeast two-hybrid system. (A) Cell growth in the absence of histidine, tryptophan, and leucine. (B) Cell growth in the absence of tryptophan and leucine. Growth in histidine-deficient medium represents selective conditions for protein-protein interaction.

We first assessed TMV MP^1–50^ and SYTA interactions using the yeast two-hybrid system, in which protein interaction is detected based on histidine prototrophy. As shown in Fig. 1A, MP^1–50^ interacted with SYTA. SYTA also interacted with the MP^1–50(V4A)^ missense mutant. However, SYTA did not interact with the missense mutant MP^1–50(F14A)^, in which the conserved Phe-14 was mutated, nor did it interact with TMV MP^Δ1–50^, which lacks the N-terminal TMV MP 50 amino acid residues that contain the PLS (Fig. 1A). In control experiments, none of the tested protein combinations interfered with cell growth under nonselective conditions (i.e., in the presence of histidine) (Fig. 1B).

**FIG 2:**
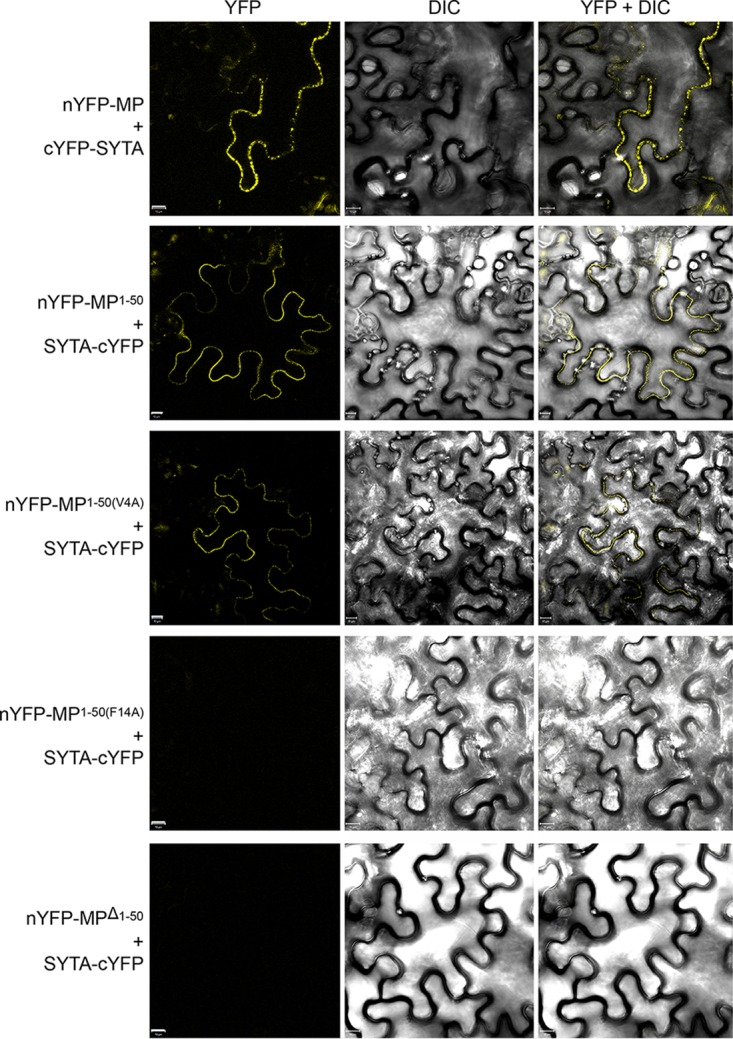
MP^1–50^-SYTA interaction in living plant cells. Protein interaction was analyzed by BiFC in N. benthamiana leaves agroinfiltrated with the tested combinations (1:1 wt/wt ratio) of expression constructs. YFP signal is in yellow; plastid autofluorescence was filtered out. Fluorescence images are single confocal sections. DIC, differential inference contrast. Scale bars = 10 µm.

**FIG 3:**
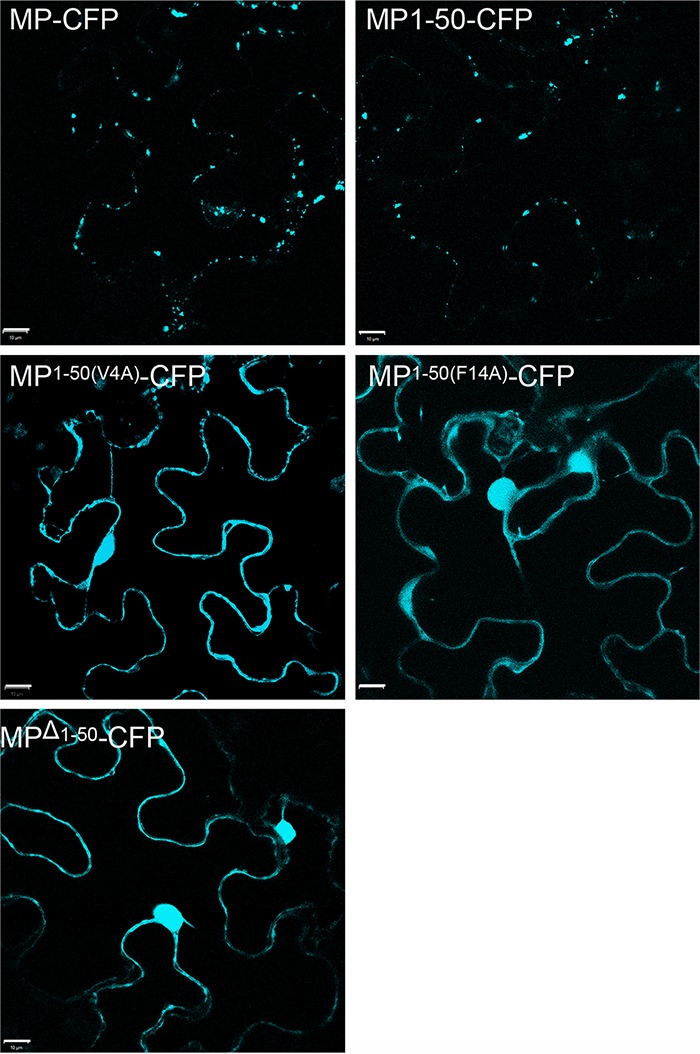
Pd targeting of MP, MP^1–50^, MP^1–50(V4A)^, PLS^F14A^, and MP^Δ1–50^. Subcellular localization of MP-CFP, MP^1–50^-CFP, MP^1–50^(V4A)-CFP, MP^1–50^(F14A)-CFP, and MPΔ^1–50^-CFP was analyzed in microbombarded wild-type Col-0 *Arabidopsis* leaves. CFP signal is blue; plastid autofluorescence was filtered out. Images are single confocal sections. Scale bars = 10 µm.

We next used bimolecular fluorescence complementation (BiFC) (21, 22) to confirm the MP^1–50^-SYTA interaction *in planta*. In this approach, a molecule of yellow fluorescent protein (YFP) is separated into two parts, N-terminal (nYFP) and C-terminal (cYFP), neither of which fluoresces when expressed alone, but the fluorescence is restored when nYFP and cYFP are brought together as fusions with interacting proteins (21, 22). BiFC analyses have shown that TVCV MP interacts with SYTA early in virus infection to recruit SYTA to Pd (14). These studies further suggest that TVCV MP and SYTA transiently interact in the absence of infection (14). The free amino terminus of TMV MP is required for it to be transported to Pd (13). Thus, in order to show stable interaction of TMV MP^1–50^ with SYTA, which is normally located at ER-PM contact sites, we blocked the amino terminus of full-length MP and MP^1–50^ by fusing each to the nYFP BiFC reporter. Figure 2 shows that, as expected (14, 15), nYFP-tagged full-length TMV MP interacted with SYTA-cYFP in plant cells, with the interacting proteins accumulating along the plasma membrane of the cell at the SYTA-marked ER-PM contact sites. Similarly, we found that SYTA-cYFP interacted with both nYFP-MP^1–50^ and nYFP-MP^1–50(V4A)^ (Fig. 2). Fitting with this, nYFP-MP^Δ1–50^ did not interact with SYTA-cYFP: we did not detect a fluorescent signal when we coexpressed SYTA-cYFP with nYFP-MP^Δ1–50^ (Fig. 2), even though cyan fluorescent protein (CFP)-MP^Δ1–50^, when transiently expressed, does stably accumulate in plant cells (13). We also did not detect a signal when we coexpressed SYTA-cYFP with nYFP-MP^1–50(F14A)^ (Fig. 2), again indicating lack of interaction, although reduced stable autofluorescence of this MP fusion construct cannot be ruled out. Overall, our BiFC results closely paralleled those obtained in the yeast two-hybrid system (Fig. 1A). Consistent with our previous findings in *Nicotiana benthamiana* (13), MP-CFP and MP^1–50^-CFP, each tagged at their carboxy terminus with CFP and transiently expressed in *Arabidopsis* Col-0 leaf cells, were found to accumulate at Pd, identified by their diagnostic punctate pattern along the cell wall (10, 13, 23–28) (Fig. 3). In contrast, MP^1–50^(V4A)-CFP, MP^1–50^(F14A)-CFP, and MPΔ^1–50^-CFP displayed a nucleocytoplasmic pattern of subcellular distribution (Fig. 3). Collectively, our data show that SYTA interacted with TMV MP^1–50^, which contains the PLS, and further suggested that residue Phe-14 within the PLS may be a critical site for both this interaction and Pd targeting.

### The Pd targeting activity of TMV MP PLS requires SYTA.

To better understand the involvement of SYTA in the ability of the PLS to target TMV MP to Pd, we used the well-characterized *syta-1* line of *Arabidopsis* Col-0, a transfer DNA (T-DNA) insertional knockdown *SYTA* mutant (14, 15). This homozygous line accumulates full-length SYTA protein to ~10% of the levels found in wild-type Col-0 (15), yet the *syta-1* plants have normal fertility, and they do not develop dramatic phenotypes under either long- or short-day conditions (15). Previous studies of the *Tobamovirus* TVCV using this *syta-1* knockdown line demonstrate that SYTA at ER-PM contact sites is required for TVCV MP to accumulate in Pd and further show that TVCV MP recruits SYTA to Pd for virus replication and movement (14). In these studies, TVCV MP, when transiently expressed, accumulated in Pd in *syta-1* leaf cells to ~50% of the levels found in wild-type Col-0 plants at 24 h postbombardment, whereas the Pd levels of PDLP1, which unlike *Tobamovirus* MPs traffics to Pd through the secretory pathway (29), were the same in both lines (14). We therefore transiently expressed TMV MP-CFP or MP^1–50^-CFP in leaf cells of wild-type Col-0 and mutant *syta-1* plants. We also expressed DsRed2-tagged PDCB1, a Pd-localized glycosylphosphatidylinositol (GPI)-anchored membrane protein that presumably reaches Pd via the secretory pathway (30). We quantified our results using integrated density measurements.

**FIG 4:**
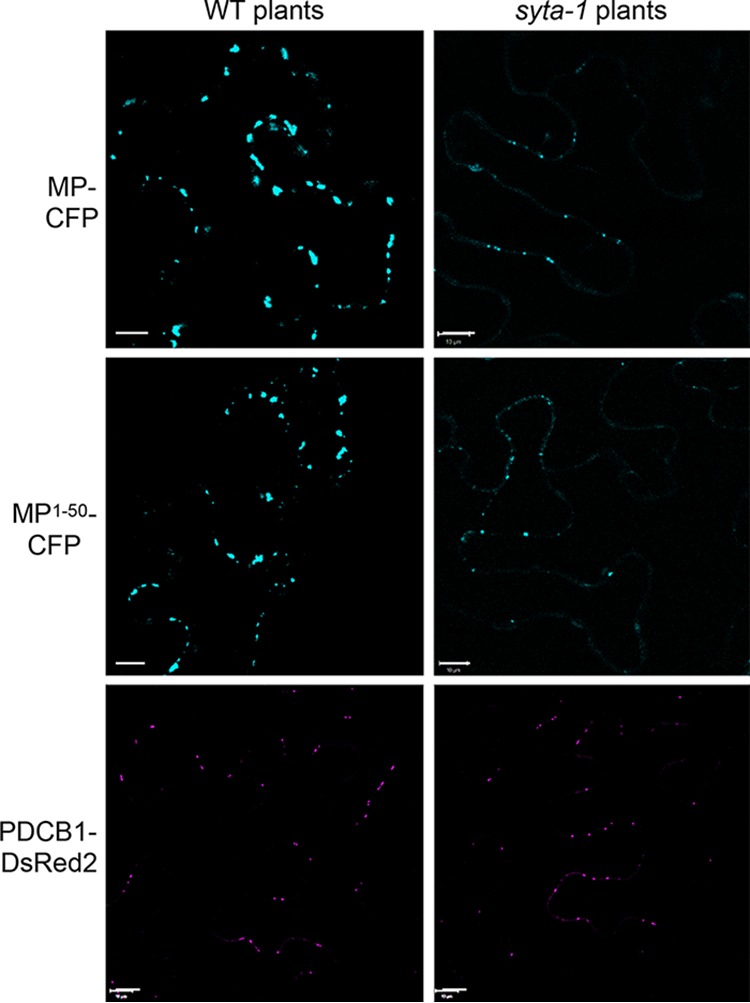
Pd targeting of MP, MP^1–50^, and PDCB1 in the wild-type and *syta-1* mutant plants. Subcellular localization of MP-CFP, MP^1–50^-CFP, and PDCB1-DsRed2 was analyzed in microbombarded leaves of the wild-type (WT) Col-0 and mutant *syta-1 Arabidopsis* plants. CFP signal is blue; DsRed2 signal is in red. Plastid autofluorescence was filtered out. Images are single confocal sections. Scale bars = 10 µm.

**FIG 5:**
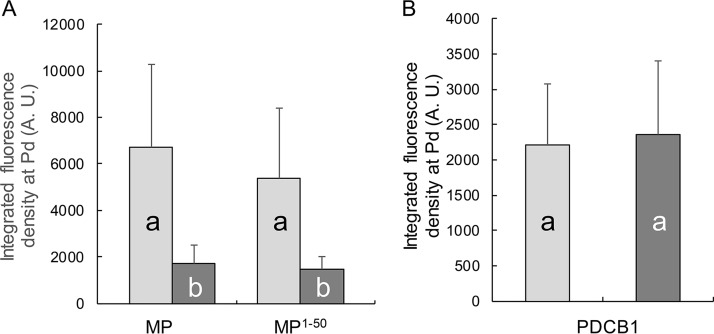
Quantification of Pd targeting of MP, MP^1–50^, and PDCB1 in the wild-type and *syta-1* mutant plants. (A) Pd targeting of MP-CFP and MP^1–50^-CFP. (B) Pd targeting of PDCB1-DsRed2. Protein accumulation at Pd was assessed by integrated density measurements of the data obtained as described in Fig. 4. Error bars represent standard error of the mean (SEM) from three biological replicates. Light and dark gray bars represent wild-type Col-0 and mutant *syta-1 Arabidopsis* plants, respectively. Differences in Pd accumulation indicated by different letters are statistically significant (*P* values <0.001), and those by the same letter are not statistically significant. A. U., arbitrary units.

As shown in Fig. 4, both TMV MP-CFP and MP^1–50^-CFP, which contains the PLS, accumulated at Pd in wild-type Col-0 and in *syta-1* plants. However, as found for TVCV MP (14), both TMV MP-CFP and MP^1–50^-CFP accumulated to lower levels at Pd in *syta-1* Col-0 compared to wild-type Col-0. In contrast, PDCB1-DsRed2 appeared to accumulate at Pd to comparable levels in both wild-type Col-0 and *syta-1* plants (Fig. 4). Our integrated density measurements confirmed these differences: both TMV MP-CFP and MP^1–50^-CFP accumulated at Pd in *syta-1* leaf cells to ~35%, the levels found in wild-type Col-0, whereas, as previously shown for PDLP1 (14), there was no statistical difference in the accumulation of PDCB1-DsRed2 at Pd (Fig. 5).

## DISCUSSION

The ER and endomembrane system have been shown to be necessary for TMV MP and TVCV MP to target to Pd, although neither MP traffics through the secretory pathway to reach Pd (18, 19, 31, 32). Recent studies establish that interaction with SYTA at ER-PM contact sites is required for TVCV MP to accumulate in Pd during virus infection (14). The C-terminal region of SYTA comprising the two Ca^2+^/lipid binding domains has been implicated in this interaction (15), but the TVCV MP sequence or sequences required to interact with SYTA have not been identified. We show here that TMV MP^1–50^, which contains the PLS sequence, is necessary and sufficient for TMV MP to interact with SYTA (Fig. 1 and 2). A specific point mutation in the PLS sequence that rendered it inactive for Pd targeting (13) also abolished the interaction with SYTA (Fig. 3). In addition, the accumulation of TMV MP^1–50^ at Pd was markedly reduced in the Arabidopsis syta*-1* knockdown line (Fig. 4 and 5).

SYTA localizes to ER-PM contact sites in plant cells and is required to form these sites, tethering the ER to the plasma membrane, in *Arabidopsis* (14). Studies of TVCV show that during infection, TVCV MP interacts with SYTA at ER-PM contact sites near Pd, remodeling these sites to form virus replication sites at Pd and relocate SYTA within Pd that are active in TVCV MP cell-to-cell transport (14). However, while TVCV MP (14), as well as TMV MP and their viral RNA genome cargo (reviewed in reference 33), move through Pd, SYTA itself does not move through Pd (14). In a similar manner, the TMV MP PLS acts as a bona fide targeting sequence that is not involved in subsequent protein movement through the Pd channel (13). This suggests that the interaction with SYTA most likely represents one step, and possibly the concluding step, in the Pd targeting pathway of MP. This step aims to stabilize, via the PLS-SYTA interaction, the association of MP with plasma membrane regions lining Pd. Indeed, MP^1–50^ alone, which contains the PLS, has been shown to target to Pd, but it remains associated with the plasma membrane rather than with the more distant cell wall-resident portion of Pd (13). In addition to TMV MP and TVCV MP, SYTA is involved in Pd transport and infectivity of other plant viruses in *Arabidopsis*, specifically the unrelated *Begomovirus Cabbage leaf curl virus* (CaLCuV) and the *Potyvirus Turnip mosaic virus* (TuMV) (14, 15). If and when PLSs are identified in the MPs of these viruses, it will be interesting to examine whether these sequences, analogous sequences, or perhaps distinct sequences are those recognized by SYTA.

## MATERIALS AND METHODS

### Plants.

*Nicotiana benthamiana* plants were grown on soil in an environment-controlled chamber at 22°C under a 16-h light (75 µmol photons m^−2^ s^−1^)/8-h dark cycle. The homozygous Arabidopsis thaliana Col-0 *syta-1* mutant was described previously (15). Wild-type Col-0 and *syta-1* plants were grown on soil in an environment-controlled chamber at 23°C under a 16-h light (100 µmol photons m^−2^ s^−1^)/8-h dark cycle.

### Constructs.

For subcellular localization studies, we used the binary plasmids expressing MP-CFP, MP^1–50^-CFP, MP^1–50^(V4A)-CFP, MP^1–50^(F14A)-CFP, and MPΔ^1–50^-CFP. These constructs and PDCB1-DsRed2 were previously described (13).

For BiFC constructs expressing nYFP-MP^1–50^, nYFP-MP^1–50(V4A)^, nYFP-MP^1–50(F14A)^, and nYFP-MP^Δ1–50^, each corresponding coding sequence was transferred from the original pSAT1 vector (13) into the HindIII-KpnI sites of pSAT4-nEYFP-C1 (34). To construct SYTA-cYFP, the SYTA coding sequence was PCR amplified from pBSII SK (15) and cloned into the EcoRI-KpnI sites of pSAT1-nEYFP-N1 (34).

For yeast two-hybrid constructs, LexA fusions of MP^1–50^, MP^1–50(V4A)^, MP^1–50(F14A)^, and MP^Δ1–50^ were generated by transferring the corresponding coding sequences from the pSAT1 vectors (13) into the EcoRI-PstI sites of pSTT91(TRP1+) (35). To construct the LexA-SYTA fusion, the SYTA coding sequence in pBSII SK (15) was cloned into the EcoRI-PstI sites of pGAD424(LEU2+) (Clontech; Mountain View, CA). All constructs were verified by DNA sequencing.

### Yeast two-hybrid assay.

Each test construct was introduced into Saccharomyces cerevisiae strain TAT7(L40-ura3) (36), plated at the indicated cell densities on media lacking leucine, tryptophan, and histidine, and grown for 2 days at 30°C. For control experiments, cells were plated and grown on media deficient in leucine and tryptophan. Cell growth media and conditions were as described before (37, 38). Positive interactions were detected by histidine prototrophy (39).

### Plant transient expression studies.

N. benthamiana plants at the 6- to 10 leaf-stage (6 weeks old or less, largest leaves ~6 to 8 cm in diameter) were used for agroinfiltration. The appropriate binary plasmids in Agrobacterium tumefaciens strain EHA105 (40) were grown overnight at 28°C, and cultures diluted to cell density of of 0.3 at *A*_600_ were infiltrated into the abaxial side of intact leaves of N. benthamiana immediately above the cotyledon using a 1-ml syringe as described previously (41, 42).

For biolistic delivery, each test construct (50 μg) was adsorbed onto 10 mg of 1-µm-diameter gold particles (Bio-Rad, Hercules, CA), and the leaf epidermis of 5- to 6-week-old *Arabidopsis* Col-0 was bombarded with them using a Helios gene gun system (model PDS-1000/He; Bio-Rad) at 80 to 110 lb/in^2^ pressure as described previously (10). Leaves were analyzed using confocal microscopy at 24 to 48 h postagroinfiltration or postbombardment. At least 10 plants were used for each experimental condition, and all experiments were repeated three times.

### Confocal laser-scanning microscopy.

Images were collected with a Zeiss LSM 5 Pascal laser-scanning confocal microscope. A 458- or 488-nm line from an argon ion laser was used to excite CFP and YFP, respectively, and a 543-nm line from a helium-neon ion laser was used to excite DsRed2. Image acquisition settings (laser intensity and photomultiplier tube [PMT]) were maintained between different experiments. From 100 to 120 cells were examined for each experiment.

To measure integrated density signals, all confocal images were collected using the same setting references and imported into Adobe Photoshop, and the signal of nonspecific protein aggregations was eliminated. The integrated density signal at Pd was then quantified using the ImageJ software (version 1.51, NIH) with the “analyze particles” command (http://imagej.nih.gov/ij/). The detected particle size was set to 3 to 50 px^2^, and the threshold was adjusted to 50 as previously described (14). All experiments were repeated at least three times in independent biological replicates.
